# Evaluation of convolutional neural networks for the detection of inter-breath-hold motion from a stack of cardiac short axis slice images

**DOI:** 10.1186/s12880-023-01070-x

**Published:** 2023-08-24

**Authors:** Yoon-Chul Kim, Min Woo Kim

**Affiliations:** 1 Division of Digital Healthcare, College of Software and Digital Healthcare Convergence, Yonsei University, Wonju, 26493 Gangwon-do South Korea; 2 Department of Computer Science and Engineering, Sogang University, Seoul, South Korea

**Keywords:** MRI, Heart, Medical image processing, Deep learning, Motion detection

## Abstract

**Purpose:**

This study aimed to develop and validate a deep learning-based method that detects inter-breath-hold motion from an estimated cardiac long axis image reconstructed from a stack of short axis cardiac cine images.

**Methods:**

Cardiac cine magnetic resonance image data from all short axis slices and 2-/3-/4-chamber long axis slices were considered for the study. Data from 740 subjects were used for model development, and data from 491 subjects were used for testing. The method utilized the slice orientation information to calculate the intersection line of a short axis plane and a long axis plane. An estimated long axis image is shown along with a long axis image as a motion-free reference image, which enables visual assessment of the inter-breath-hold motion from the estimated long axis image. The estimated long axis image was labeled as either a motion-corrupted or a motion-free image. Deep convolutional neural network (CNN) models were developed and validated using the labeled data.

**Results:**

The method was fully automatic in obtaining long axis images reformatted from a 3D stack of short axis slices and predicting the presence/absence of inter-breath-hold motion. The deep CNN model with EfficientNet-B0 as a feature extractor was effective at motion detection with an area under the receiver operating characteristic (AUC) curve of 0.87 for the testing data.

**Conclusion:**

The proposed method can automatically assess inter-breath-hold motion in a stack of cardiac cine short axis slices. The method can help prospectively reacquire problematic short axis slices or retrospectively correct motion.

**Supplementary Information:**

The online version contains supplementary material available at 10.1186/s12880-023-01070-x.

## Introduction

**Fig. 1:**
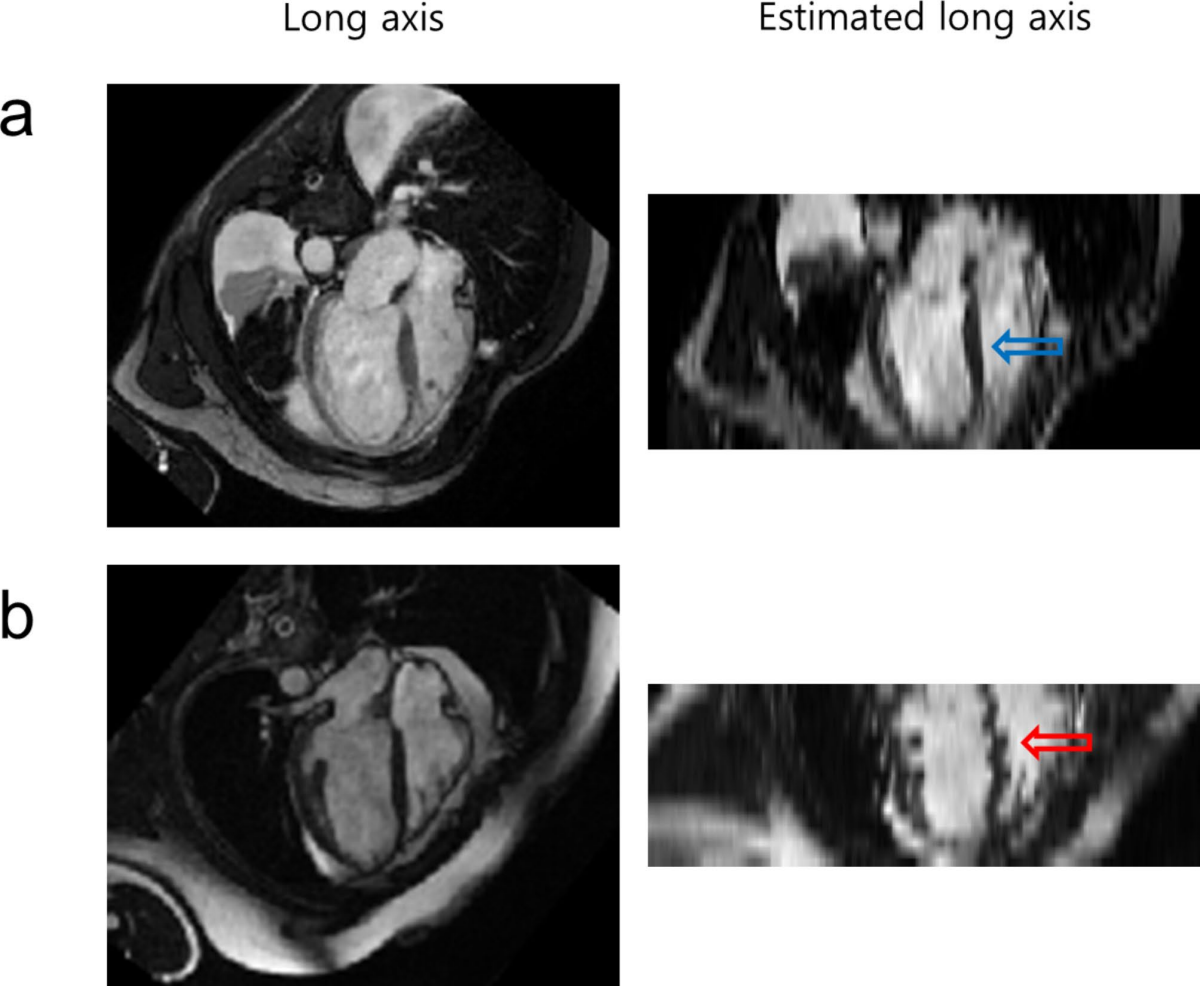
Examples of **(a)** no inter-breath-hold motion (blue arrow) and **(b)** inter-breath-hold motion (red arrow) in long axis slice images estimated from a stack of short axis slice images

Cardiovascular disease is the primary cause of death in developed countries and includes heart failure, arrhythmia, valve disease, and coronary artery disease [1]. Heart failure develops when the heart does not pump the blood sufficiently to the body’s needs. Left ventricular (LV) ejection fraction is considered as an important biomarker for the assessment of heart failure [2]. Cardiac cine magnetic resonance imaging (MRI) enables quantification of the LV ejection fraction with high spatial resolution images [3]. Cardiac cine MRI typically requires multiple breath-holds to cover the entire LV with a stack of short axis slices, and patients may perform breath-holds in different respiratory positions, potentially leading to inconsistency in the heart location and irregularity in the ventricular septum (Fig. 1) and thus to potential inaccuracy in the LV diastolic/systolic volumes and in three-dimensional (3D) modeling of the LV [4]. Detection of misalignment of cardiac cine short axis slices is necessary for image quality assessment and for further analysis and visualization [5]. Alignment of cardiac cine short axis slices has implications for improved 3D visualization of the LV. Previous related studies investigated motion correction between short axis and long axis cine slices [6, 7] for cardiac image analysis. Swingen et al. estimated the magnitude of the misregistration of a short axis image of the heart and aligned the center of gravity of each short axis slice’s endocardial contour for breath-hold motion correction [5]. The motion correction involved iterative minimization of a cost function that includes displacements between intensity profiles of the intersected lines. A drawback of the method was large computation time. Another approach for motion correction in cardiac cine images is to delineate the LV contours manually on both long axis and short axis image planes and register all contours using a two-step iterative closest point algorithm [8]. A drawback of the method is large computation time taken to manually segment the contours, taking approximately nine minutes per case.

Deep learning has been extensively used for cardiac image analysis with the aim of automatically classifying diseases, identifying cardiovascular disease risks, segmenting regions of interest, generating high quality images, and estimating biomarker quantities [9–11]. Several deep learning methods for cardiac image classification have been demonstrated in the literature. For example, view classification in echocardiographic images with deep convolutional neural network (CNN) has been demonstrated to accurately classify five standard views (long axis, short axis, 2-chamber, 3-chamber, and 4-chamber) [12]. Also, cross-sections of the coronary arteries in coronary computed tomography (CT) angiography were used to train and validate the presence/absence of motion artifacts using deep CNN [13]. However, to the best of our knowledge, there have been no studies that demonstrate the performance of deep CNN models in classifying the presence of motion from cardiac long axis cine MRI images, which are reformatted from a 3D stack of short axis slices.

In this study, we present a deep learning-based method that classifies the presence or absence of the inter-breath-hold motion from an estimated long axis slice image. Training data are generated using a tool that enables the user to annotate the presence/absence of motion based on visual inspection of a long axis slice image reconstructed from a stack of short axis slices, along with an acquired long axis slice image as a reference. This facilitates the generation of training data for supervised learning of inter-breath-hold motion detection models. Finally, a variety of deep CNN classification models are developed using training data, and they are validated on unseen testing data.

## Methods

### Data

In the present study, we used publicly available data from the LV cardiac MRI segmentation challenge [14] (referred to as CAT) and the Kaggle 2nd Annual cardiac challenge (referred to as KAG) (https://www.kaggle.com/c/second-annual-data-science-bowl). Table 1 summarizes the numbers of subjects considered for each dataset in model development and testing. First, cardiac cine DICOM (Digital Imaging and Communications in Medicine) [15] data from all short axis slices and several 2-/3-/4-chamber long axis slices from 185 subjects were considered for the CAT dataset. Second, cardiac cine DICOM data from all short axis slices and 2-/4-chamber long axis slices from 1,046 subjects were considered for the KAG dataset. Image acquisition parameters were as follows: steady-state free precession (SSFP) sequence, slice thickness $\le$ 10 mm, inter-slice gap $\le$ 2 mm, repetition time (TR) = 30–50 ms, echo time (TE) = 1.6 ms, flip angle = 60°, field-of-view (FOV) = 360 mm, spatial resolution = 0.7031–2.0833 mm^2^ [16]. From the dynamic cine image frames of 20–30, we considered the initial time frame, which corresponds to the end-diastole.

**Table 1:** Numbers of subjects for the two datasets in model development and testing

	Dataset	
	CAT	KAG
Model development (training + validation)	90	650
Testing	95	396
Total	185	1,046

### Preprocessing

**Fig. 2:**
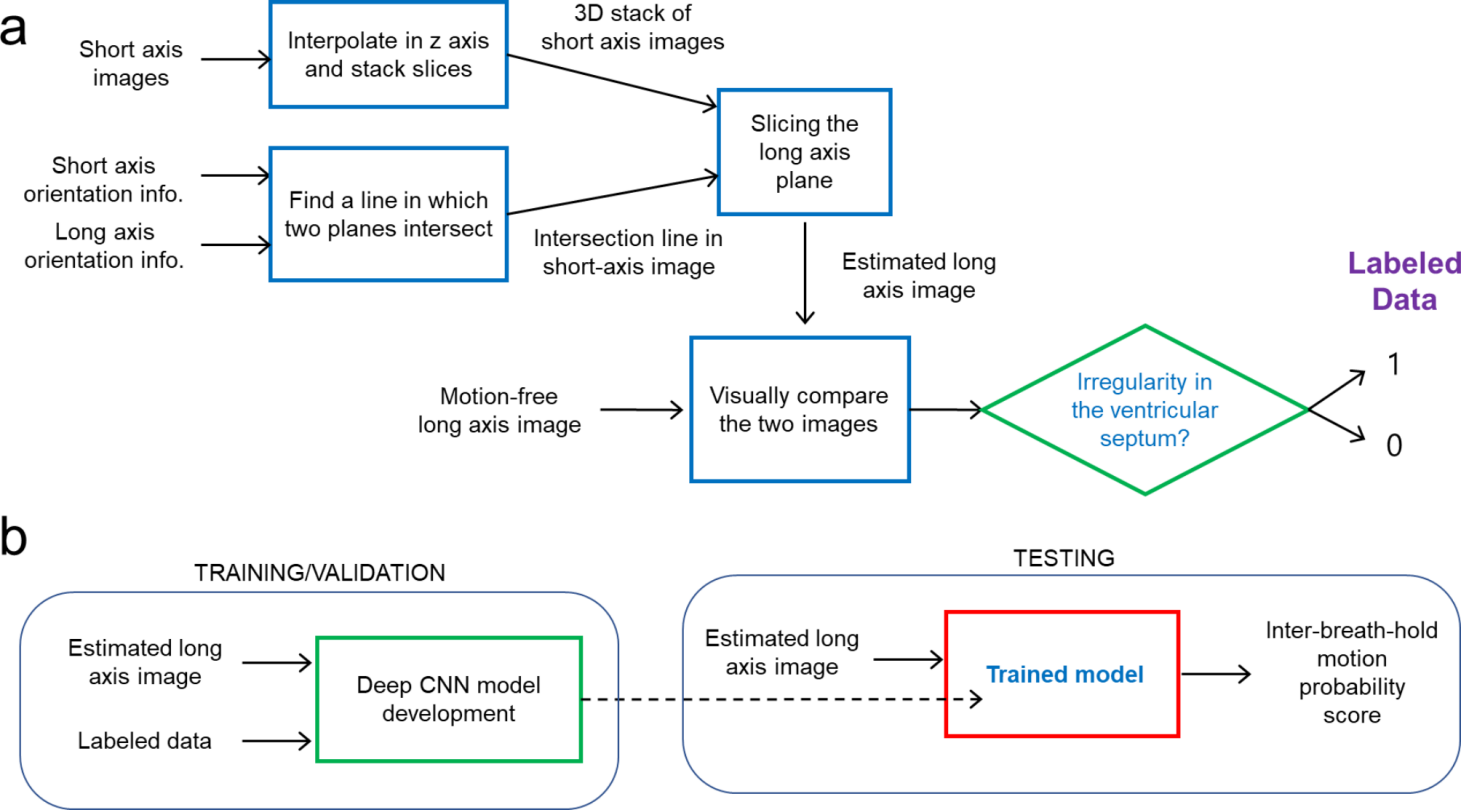
A flowchart of the presented method. **(a)** Data labeling process. **(b)** Deep CNN model development and testing process. The method was designed to automatically assess inter-breath-hold motion in cardiac short axis slices acquired during multiple breath-holds

**Fig. 3:**
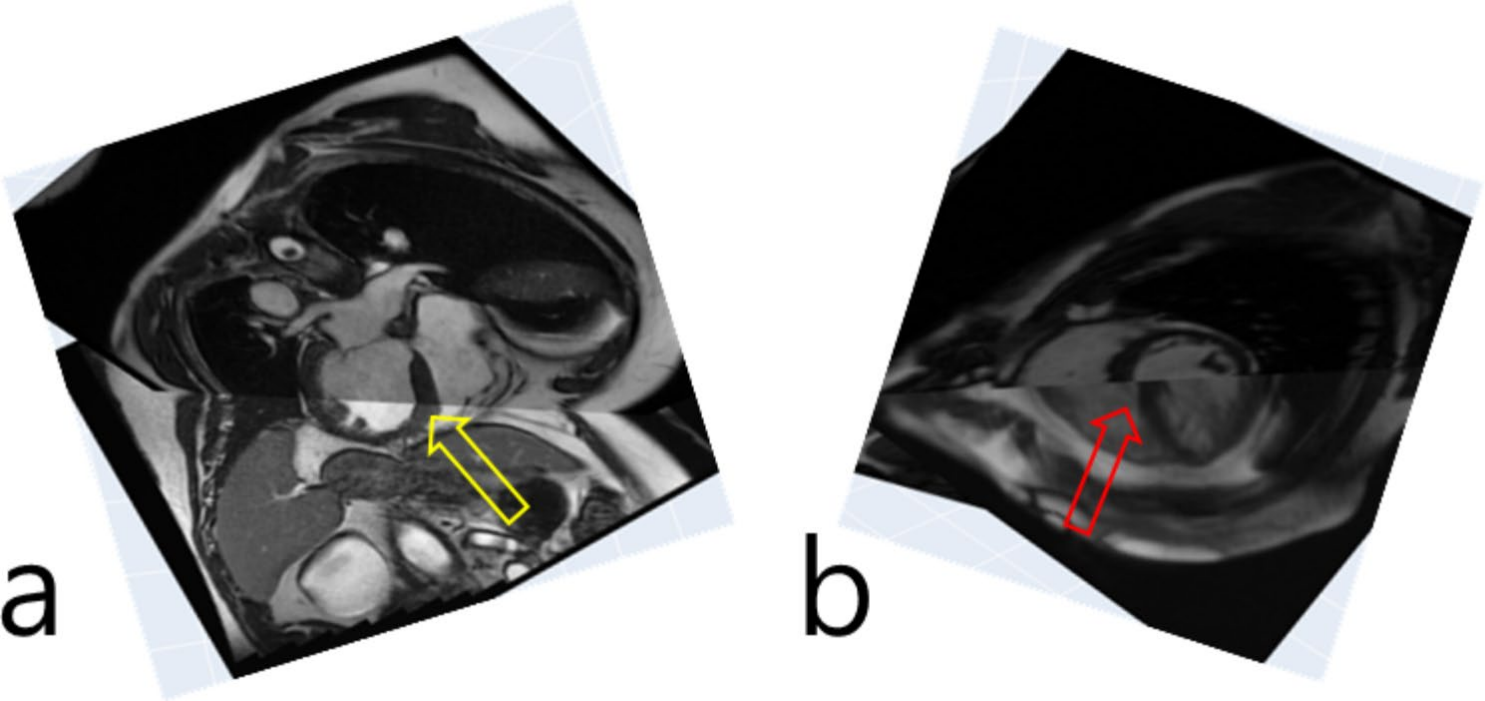
Visualization of the two orthogonal scan planes. Slice misalignment is not observed in **(a)** (yellow arrow), while it is observed in **(b)** (red arrow) in the intersecting line between the two planes

Figure 2 shows a flowchart of the current method. Figure 2a illustrates a flowchart of training data generation, while Fig. 2b shows a block diagram of deep learning model development and testing. A software tool was developed in Python 3.10. Intersecting lines were calculated based on the pixel spacing, image position, and slice orientation in the DICOM header information. A pair of the original long axis image and the same orientation view of an estimated long axis image was obtained. The estimated long axis image was reconstructed from a 3D stack of short axis images after slice reformatting based on the slice orientation information. The user interface tool shows a long axis image as a reference (the left image in Figure S1 of the Supplemental Material) and an estimated long axis image (the right image in Figure S1 of the Supplemental Material). Slice navigation bars are located above the three images to change either the long axis view or the slice number of the short axis view. The tool was effective in debugging the code for the generation of the estimated long axis image. The estimated long axis slice images and original long axis images of the same orientation were saved as .png files. In addition, as shown in Fig. 3, Plotly (v4.9.0) was used to visually check the misregistration in the intersection line between the long axis and short axis cine images in a web browser [17].

Another graphical user interface tool was developed in MATLAB (Mathworks, Inc., Natick, MA, USA) [18] to assist the manual labeling of the presence/absence of inter-breath-hold motion (Figure S2 of the Supplemental Material). The presence of inter-breath-hold motion was inferred from an irregular shape of the ventricular septum (e.g., the red arrow in Fig. 1b) or the lateral ventricular wall from an estimated long axis image. Estimated long axis images were classified as “outlier” when the images exhibit poor image quality for the interpretation of inter-breath-hold motion. The labeled results, along with the image file names, were saved as an Excel spreadsheet file.

### Deep learning

Data from a total of 1,231 subjects were considered for the training/validation/testing of deep CNN models. Image data from 740 subjects belonged to the model development group, and image data from 491 subjects belonged to the testing group. The data for model development consisted of the training data of the CAT dataset and the training and validation data of the KAG dataset. The data for the testing consisted of the validation data of the CAT dataset and the testing data of the KAG dataset. With data from the model development group, a five-fold cross validation procedure was performed to train and validate five deep CNN models. The deep CNN models were implemented in Keras [19]. The left and right parts of each estimated long axis image were cropped, and the central part of the image contained 2-, 3-, or 4-chamber view of the heart.

We compared 12 different deep learning models, which were two customized deep CNN models (one with data augmentation and the other without data augmentation) and ten transfer learning-based pre-trained CNN models (five with data augmentation and five without data augmentation). Each of the two customized deep CNN models consisted of a series of four convolution, batch normalization [20], ReLu activation, and max-pooling layers, followed by two fully connected (FC) layers. Between the FC layers, ReLu activation and dropout [21] with a rate of 0.5 layers were included. The transfer learning-based models had EfficientNet-B0 [22], MobileNet [23], NASNetMobile [24], ResNet50 [25], and VGG16 [26] as baseline models for feature extraction [27]. These baseline models were pre-trained with ImageNet data [28], and their weight parameters were frozen for our model development. The extracted features went through global average pooling [29] followed by a fully connected layer. The output had two classes of motion and no-motion. A binary cross-entropy function was used with the Adam optimizer [30]. Since the data were imbalanced between the motion and no-motion classes, we used the Scikit-learn’s class_weight.compute_class_weight function to compute the class weights and then applied the weights to the loss function [31].

Each input image was resampled to the dimensions of 96 × 128 × 3 for the customized deep CNN model. For the transfer learning-based models, each input image was resampled to the dimensions of 224 × 224 × 3, which is the default setting for input image dimensions in the Keras deep learning library (https://keras.io/api/applications/). The three RGB channels were replicated with the same gray scale image. After a session of trial and error with different values of the learning rate of the Adam optimizer, the learning rate was set to 0.00001 for the customized deep CNN model and 0.0001 for the transfer learning-based models. The batch size was set to 4 for the customized deep CNN model and 2 for the transfer learning-based models. The training and validation were performed for 50 epochs, and the model parameters were saved at every epoch. For each fold, we chose the epoch number which showed the maximum value of validation accuracy.

### Evaluation

We implemented the methods on a Windows PC (AMD Ryzen 7 1800X Eight-Core Processor, 16 GB RAM, and NVIDIA GeForce GTX 1080 with 8 GB memory). For either the customized deep CNN or the transfer learning-based model, we evaluated the performance of classification accuracy using five-fold cross validation. Two different image augmentation schemes were considered: (1) no data augmentation performed in the training data (NoAug) and (2) horizontal flip performed to double the training data (Aug w/ flipLR). The numbers of images for each fold and each augmentation scheme are listed in Table 2. For each method, each of the five trained models predicted the inter-breath-hold motion probability score in each image. The final probability score was calculated by averaging the probability scores across the five cross-validated deep CNN models. Using the scikit-learn library [31], we compared the area under the receiver operating characteristic curve (AUC), F1-score, precision, recall, and accuracy values among the 12 deep learning models.

**Table 2:** Number of images in each fold for training and validation data

		Inter-breath-hold motion	Fold 1 (n = 148)	Fold 2 (n = 148)	Fold 3 (n = 148)	Fold 4 (n = 148)	Fold 5 (n = 148)
			No. of images	No. of images	No. of images	No. of images	No. of images
Training	No Aug	Yes	307	321	319	333	312
		No	938	939	927	915	921
	Aug w/ flipLR	Yes	614	642	638	666	624
		No	1,876	1,878	1,854	1,830	1,842
Validation		Yes	91	77	79	65	86
		No	222	221	233	245	239

## Results

The presented tool provided automatic generation of estimated long axis images from a 3D stack of short axis slices. The publicly available cardiac cine MRI data originally consisted of 200 subjects in the CAT dataset and 1,140 subjects in the KAG dataset. Among the 1,340 subjects’ data, data from 109 subjects were not considered for this study due to poor data quality or run-time errors. Poor data quality included long axis slice images too dark to evaluate and images appearing out of the LV region of interest. Run-time errors included image dimension mismatch among the short axis slice images and errors occurring during slice reformation. For example, the dimension mismatch error occurred because some short axis slices had the dimensions of 256 × 192, while other short axis slices had the dimensions of 192 × 256. For the CAT dataset, the numbers of acquired long axis slices were different for each subject. Most of the subjects had three (45%) or four (29%) long axis slices. For the KAG dataset, all subjects’ data had two long axis slices (i.e., 2-chamber and 4-chamber views). A total of 2,629 long axis images were labeled as either motion or no-motion, and 745 (28.3%) of these long axis images were labeled as motion.

Five-fold cross validation results of the customized deep CNN model and transfer learning-based model are shown in Figures S3 and S4 of the Supplemental Material, respectively. Training accuracy of the customized deep CNN models was close to 1.0 at epoch 40–50 and was higher than that of the transfer learning-based models in all folds. The overfitting issue may be due to the fact that the number of model parameters in the customized models is higher than that in the transfer learning-based models. From the validation accuracy plots in Figures S3 and S4, it is shown that the validation accuracy of the customized deep CNN models has a wider variation across the five folds than that of the transfer learning-based models.

**Fig. 4:**
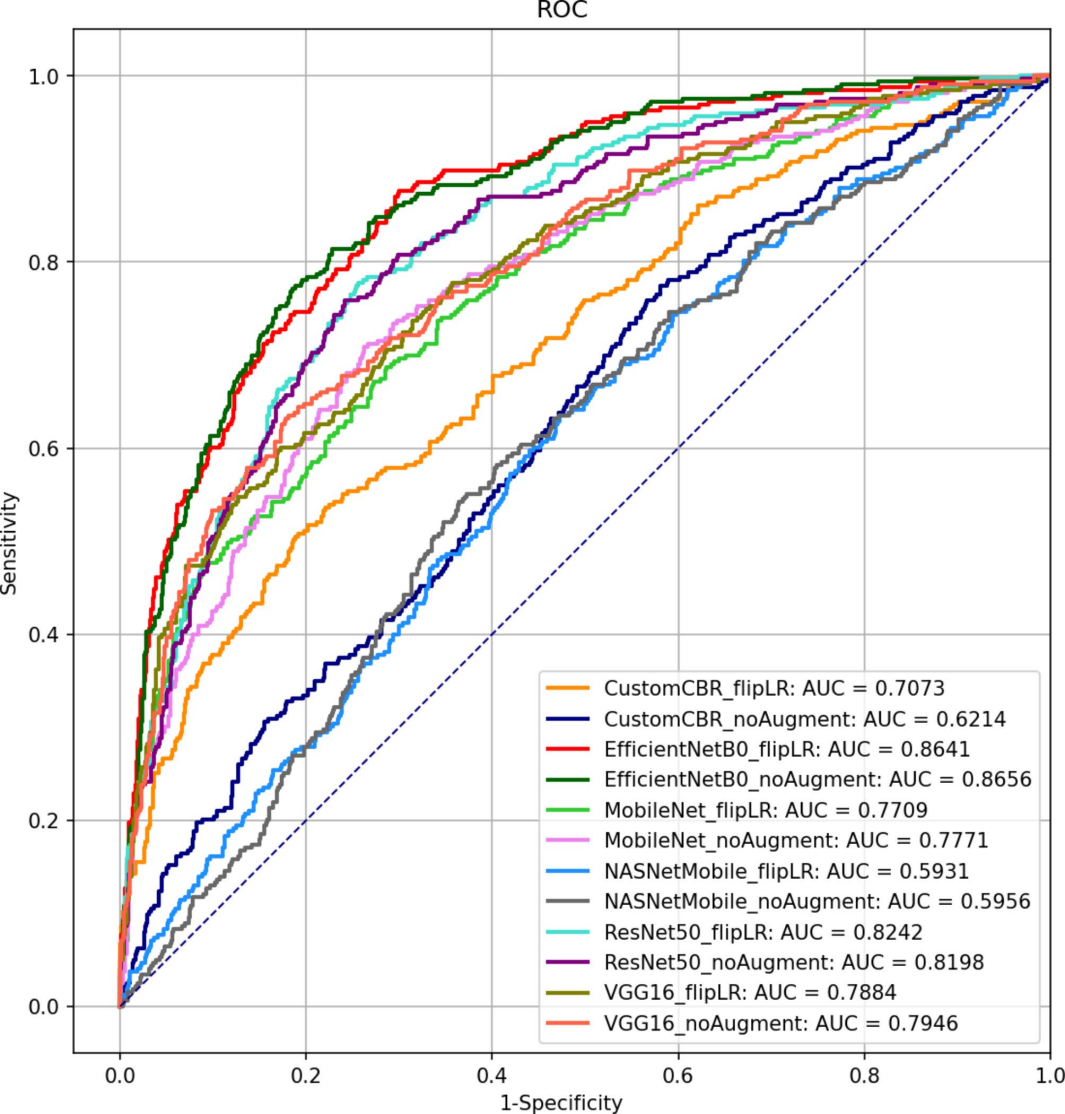
Comparison of the AUCs when evaluating the deep CNN models on the testing data

The prediction performance evaluation results of the 12 different deep learning models are shown in Table 3. The data augmentation significantly improved the AUC, F1-score, precision, recall, and accuracy scores in the customized deep CNN model. For example, the accuracy score changed from 0.5252 to 0.7168 in the customized deep learning model, suggesting that other additional augmentation schemes including random image rotation and translation may help improve prediction performance. This is expected because the model showed severe overfitting in the training and validation learning curves (Figure S3). Meanwhile, the data augmentation did not help improve the scores in the transfer learning-based models. The transfer learning-based model with EfficientNet-B0 as a feature extractor and no data augmentation resulted in the highest scores in the AUC, F1-score, precision, and accuracy metrics. The transfer learning-based model with EfficientNet-B0 as a feature extractor and data augmentation resulted in the highest score in the recall metric. The pre-trained EfficientNet-B0 model may have convolutional filters that are effective at extracting features that are relevant to the irregularity in the myocardial wall. Overall, the transfer learning-based model with NASNetMobile resulted in the lowest scores. Figure 4 shows the receiver operating characteristic (ROC) curves for the 12 deep learning models. It indicates that EfficientNet-B0 and ResNet50 models are relatively higher in AUC values than the other models.

**Table 3:** Results of motion detection. The boldface indicates the highest score among the methods

Neural network model	Data augmentation	AUC^a^	F1-score	Precision	Recall	Accuracy
Customized deep CNN with four CBR^b^ layers	No Aug	0.6214	0.4950	0.3646	0.7709	0.5252
	Aug w/ flipLR	0.7073	0.5183	0.5327	0.5046	0.7168
EfficientNet-B0	No Aug	**0.8656**	**0.6940**	**0.6065**	0.8111	**0.7841**
	Aug w/ flipLR	0.8641	0.6803	0.5573	**0.8731**	0.7523
MobileNet	No Aug	0.7771	0.6086	0.5366	0.7028	0.7271
	Aug w/ flipLR	0.7709	0.5831	0.5080	0.6842	0.7047
NASNetMobile	No Aug	0.5956	0.4550	0.3890	0.5480	0.6037
	Aug w/ flipLR	0.5931	0.4636	0.3662	0.6316	0.5589
ResNet50	No Aug	0.8198	0.6533	0.5755	0.7554	0.7579
	Aug w/ flipLR	0.8242	0.6545	0.5669	0.7740	0.7533
VGG16	No Aug	0.7946	0.6163	0.6018	0.6316	0.7626
	Aug w/ flipLR	0.7884	0.5984	0.5994	0.5975	0.7579

^a^AUC: area under receiver operating characteristic (ROC) curve

^b^CBR: convolution, batch normalization, and ReLu layers

**Fig. 5:**
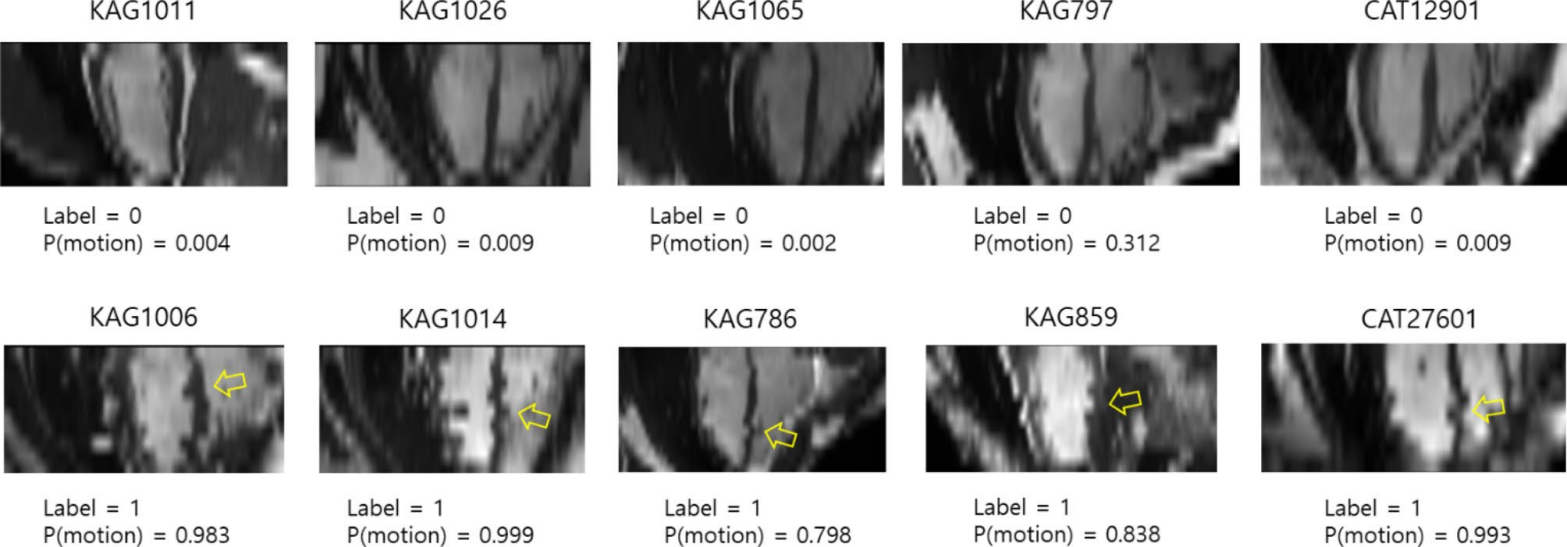
Representative examples of correct predictions in the estimated long axis images. (Top row) Examples of no inter-breath-hold motion. (Bottom row) Examples of inter-breath-hold motion. P(motion) indicates a probability score of predicting the presence of inter-breath-hold motion when using the model of the EfficientNet-B0 as a feature extractor without data augmentation

Figure 5 shows representative examples of correct deep learning predictions in the estimated long axis images. The top row shows images labeled as no-motion, while the bottom row shows images labeled as motion. P(motion) indicates a probability score of the inter-breath-hold motion. The transfer learning-based model with EfficientNet-B0 as a feature extractor without data augmentation was used for motion prediction. The irregularity of the ventricular septum or the ventricular wall in the lateral region is prominent (the yellow arrow) when compared with the ventricular wall of the estimated long axis images in the top row of the figure.

**Fig. 6:**
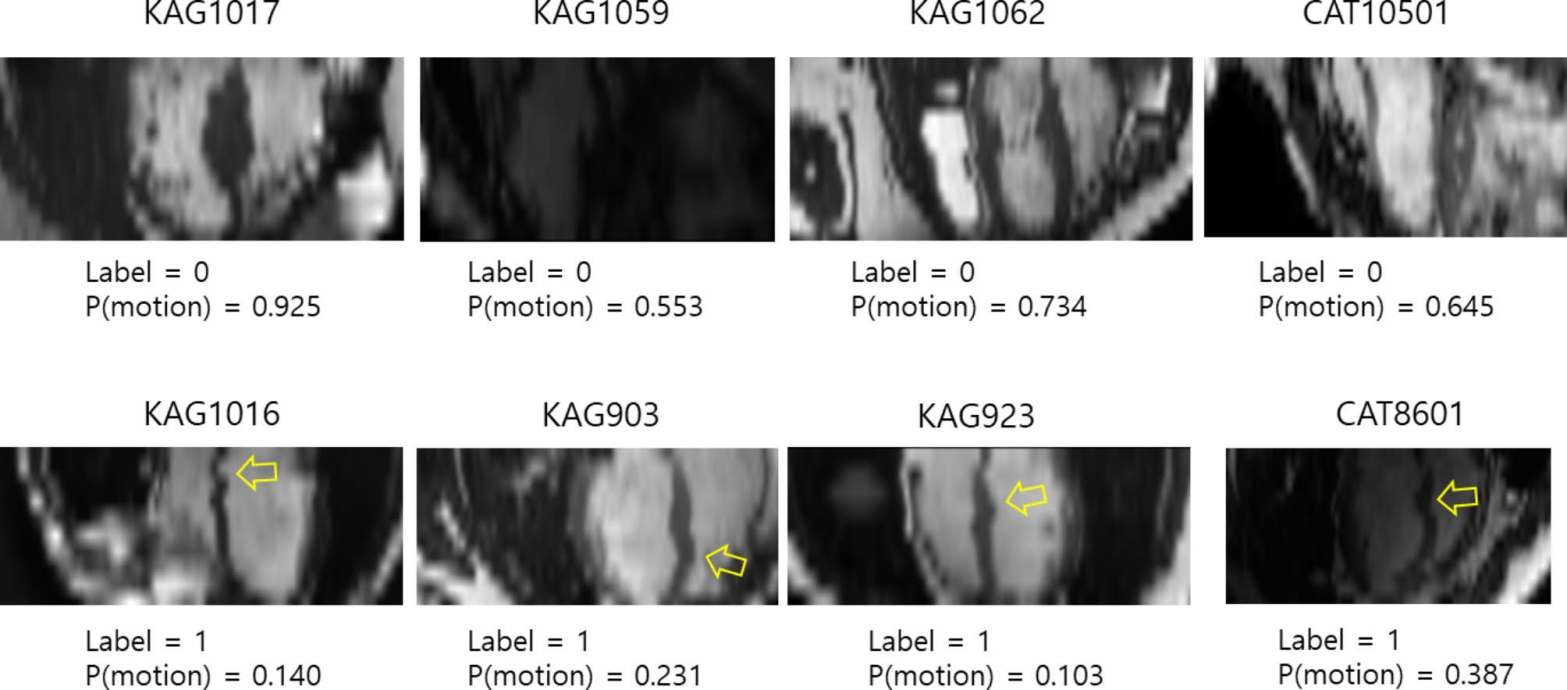
Representative examples of incorrect predictions in the estimated long axis images. (Top row) Examples of no inter-breath-hold motion. (Bottom row) Examples of inter-breath-hold motion. P(motion) indicates a probability score of predicting the presence of inter-breath-hold motion when using the model of the EfficientNet-B0 as a feature extractor without data augmentation

Figure 6 shows representative examples of incorrect deep learning predictions in the estimated long axis images. The ‘KAG1017’ image was incorrectly predicted as motion. This may be due to the thickened myocardial wall in the septum, which is a rare case in the data. The ‘KAG1059’ image was incorrectly predicted. The dark image appearance may have affected the incorrect prediction result. The ‘KAG1062’ image shows the incorrect prediction result. The bright regions close to the lateral myocardial wall may influence the incorrect prediction. The ‘KAG1016,’ ‘KAG903,’ ‘KAG923,’ and ‘CAT8601’ images all have partly tortuous myocardial bands in a few of the short-axis slices indicated by the yellow arrows in Fig. 6, suggesting inter-breath-hold motion. All of these images were incorrectly predicted as no-motion.

## Discussion

The current study demonstrates the feasibility of deep CNN models to automatically detect inter-breath-hold motion from estimated long axis slice images reformatted from a 3D stack of short axis slices. Routine cardiac cine MRI typically acquires 2-chamber, 3-chamber, and 4-chamber orientation long axis images along with the stack of short axis images with 10–15 slices that range from the apex to the basal level of the LV. This ultimately leads to up to 10–15 repetitions of breath-holds for short-axis slice imaging, which may result in inter-breath-hold motion in certain slices of the short axis slice imaging. In our study, inter-breath-hold motion was noted in approximately 28% of the subjects. Cardiac cine MRI data acquisition protocols used to collect the datasets do not seem to have detected severe inter-breath-hold motion. The inter-breath-hold motion detection method may be helpful for early detection of motion while scanning a stack of short axis cine slices. The early detection of motion may be used to suggest MRI scanner operators or MRI technicians to redo a scan. Alternatively, one can consider retrospective motion correction, but in case of severe patient motion involving both in-plane and through-plane motion, it would be very challenging to correct for the problematic slices. It may be more appropriate to discard a certain portion of the data that has been affected by severe motion.

Bright signals from the subcutaneous fat made the LV and myocardium appear very dark in certain estimated long axis images. This may have adversely affected the accuracy of motion detection. Enhancement of image contrast in the LV and myocardium can be performed during image preprocessing. Development of an image preprocessing algorithm that automatically improves the blood/myocardium contrast remains as future work. Meanwhile, it is interesting to note that the prediction accuracy of the transfer learning-based models was not highly improved with data augmentation. This may be due to the fact that the model capacity is small in the transfer learning-based models, which have a relatively small number of features (1,000 ~ 2,000) after global average pooling [32]. Fine tuning with data augmentation may help improve prediction performance [33].

The current study did not pursue retrospective motion correction. In the literature, there are a few approaches for motion correction in a stack of short axis slice images. Motion correction is based on image registration where a cost function that includes intensity displacements in the intersected lines between a long axis image and a short axis image is minimized iteratively [4, 7, 34]. A drawback of the registration-based iterative methods is their long computational time. Liew et al. reported that registration of all 20 cardiac phases took approximately 4.7 h [34]. Meanwhile, our method relied on deep CNN for motion detection. Our method, which involves the generation of estimated long axis image and the prediction of motion using deep CNN, took approximately one minute. It is relatively faster than the registration-based method, and thus it is well suited to prospective motion detection and subsequent re-scanning for motion-free short axis slice acquisition.

The current study has several limitations. First, we did not consider comparing a variety of deep CNN models with different values of learning rate. Second, it was difficult to label the “gray zone” images. For example, a certain proportion of estimated long axis images had several pixel shifts in the ventricular wall, and it was difficult to decide whether the image should be labeled as motion or no-motion. It may be more appropriate to define more than two classes, such as severe motion, slight motion, and no-motion. Third, manual labeling was performed by one expert. Although this study is a proof of concept, assessment of inter-rater or intra-rater agreement would be desirable. Fourth, this study considered the diastolic frame only. Since cardiac cine data have a temporal dimension, it would be interesting to compare the performance with all frames or a subset of the frames (e.g., end-systolic and end-diastolic frames).

## Conclusion

The presented method enabled automatic assessment of inter-breath-hold motion from a long axis slice image reformatted from a 3D stack of short axis slices. We demonstrated the feasibility of a deep CNN model, especially a transfer learning-based model, to detect inter-breath-hold motion, and this approach may help MRI operators consider rescanning patients immediately when inter-breath-hold motion is detected.

### Electronic supplementary material

Below is the link to the electronic supplementary material.

Supplementary Material 1

## Data Availability

Data in the present study were obtained from publicly available data from the Cardiac Atlas Project (http://www.cardiacatlas.org/challenges/lv-segmentation-challenge/) and from the Kaggle Second Annual Data Science Bowl (https://www.kaggle.com/c/second-annual-data-science-bowl). The datasets used and/or analyzed during the current study are available from the corresponding author on reasonable request. The code is available at https://github.com/prime52/LAmotion.

## Abbreviations

LV: Left ventricle
MRI: Magnetic resonance imaging
3D: Three-dimensional
CNN: Convolutional neural network
CT: Computed tomography
DICOM: Digital imaging and communications in medicine
SSFP: steady-state free precession
TR: Repetition time
TE: Echo time
FOV: Field of view
FC: Fully connected
RGB: Red green blue
PC: Personal computer
RAM: Random access memory
AUC: Area under the receiver operating characteristic curve
ROC: Receiver operating characteristic

## References

[1] Glovaci D, Fan W, Wong ND. Epidemiology of diabetes Mellitus and Cardiovascular Disease. Curr Cardiol Rep. p. 21, Mar 4 2019;21(4). 10.1007/s11886-019-1107-y. 10.1007/s11886-019-1107-y 30828746

[2] Groenewegen A, Rutten FH, Mosterd A, Hoes AW. “Epidemiology of heart failure,“ *Eur J Heart Fail*, vol. 22, no. 8, pp. 1342–1356, Aug 2020, doi: 10.1002/ejhf.1858. 10.1002/ejhf.1858 PMC7540043 32483830

[3] Schulz-Menger J, et al. Standardized image interpretation and post-processing in cardiovascular magnetic resonance – 2020 update: Society for Cardiovascular magnetic resonance (SCMR): Board of Trustees Task Force on standardized post-processing. J Cardiovasc Magn Reson. Mar 12 2020;22(1):19. 10.1186/s12968-020-00610-6. 10.1186/s12968-020-00610-6 PMC7066763 32160925

[4] Slomka PJ et al. “Patient motion correction for multiplanar, multi-breath-hold cardiac cine MR imaging,“ *J Magn Reson Imaging*, vol. 25, no. 5, pp. 965 – 73, May 2007, doi: 10.1002/jmri.20909. 10.1002/jmri.20909 17457798

[5] Swingen C, Seethamraju RT, Jerosch-Herold M. An approach to the three-dimensional display of left ventricular function and viability using MRI. Int J Cardiovasc Imaging. Aug 2003;19(4):325–36. 10.1023/a:1025450211508. 10.1023/a:1025450211508 14598902

[6] Carminati MC, Maffessanti F, Caiani EG. Nearly automated motion artifacts correction between multi breath-hold short-axis and long-axis cine CMR images. Comput Biol Med. Mar 2014;46:42–50. 10.1016/j.compbiomed.2013.12.013. 10.1016/j.compbiomed.2013.12.013 24529204

[7] Elen A et al. “Automatic 3-D breath-hold related motion correction of dynamic multislice MRI,“ *IEEE Trans Med Imaging*, vol. 29, no. 3, pp. 868 – 78, Mar 2010, doi: 10.1109/TMI.2009.2039145. 10.1109/TMI.2009.2039145 20199921

[8] Wan M (2016). Correcting motion in multiplanar cardiac magnetic resonance images. Biomed Eng Online.

[9] Litjens G et al. “State-of-the-Art Deep Learning in Cardiovascular Image Analysis,“ *JACC Cardiovasc Imaging*, vol. 12, no. 8 Pt 1, pp. 1549–1565, Aug 2019, doi: 10.1016/j.jcmg.2019.06.009. 10.1016/j.jcmg.2019.06.009 31395244

[10] Johnson KW et al. “Artificial Intelligence in Cardiology,“ *J Am Coll Cardiol*, vol. 71, no. 23, pp. 2668–2679, Jun 12 2018, doi: 10.1016/j.jacc.2018.03.521. 10.1016/j.jacc.2018.03.521 29880128

[11] Seetharam K, Brito D, Farjo PD, Sengupta PP (2020). The role of artificial intelligence in cardiovascular imaging: state of the art review. Front Cardiovasc Med.

[12] Kusunose K, Haga A, Inoue M, Fukuda D, Yamada H, Sata M. “Clinically Feasible and Accurate View Classification of Echocardiographic Images Using Deep Learning,“ *Biomolecules*, vol. 10, no. 5, Apr 25 2020, doi: 10.3390/biom10050665. 10.3390/biom10050665 PMC7277840 32344829

[13] Lossau T, et al. Motion artifact recognition and quantification in coronary CT angiography using convolutional neural networks. Med Image Anal. Feb 2019;52:68–79. 10.1016/j.media.2018.11.003. 10.1016/j.media.2018.11.003 30471464

[14] Fonseca CG et al. “The Cardiac Atlas Project–an imaging database for computational modeling and statistical atlases of the heart,“ *Bioinformatics*, vol. 27, no. 16, pp. 2288-95, Aug 15 2011, doi: 10.1093/bioinformatics/btr360. 10.1093/bioinformatics/btr360 PMC3150036 21737439

[15] Mildenberger P, Eichelberg M, Martin E (2002). Introduction to the DICOM standard. Eur Radiol.

[16] Dangi S, Linte CA, Yaniv Z. A distance map regularized CNN for cardiac cine MR image segmentation. Med Phys. Dec 2019;46(12):5637–51. 10.1002/mp.13853. 10.1002/mp.13853 PMC7372294 31598971

[17] Plotly Technologies Inc. Plotly, charting tool for online collaborative data science. Montréal, QC; 2015.

[18] MATLAB. Version 9.12.0 (R2022a). The MathWorks Inc.; 2022.

[19] Chollet F. “Keras: The python deep learning library,“ *Astrophysics source code library*, p. ascl: 1806.022, 2018.

[20] Ioffe S, Szegedy C. “Batch normalization: Accelerating deep network training by reducing internal covariate shift,“ in *International Conference on Machine Learning*, 2015: PMLR, pp. 448–456.

[21] Srivastava N, Hinton G, Krizhevsky A, Sutskever I, Salakhutdinov R (2014). Dropout: a simple way to prevent neural networks from overfitting. J Mach Learn Res.

[22] Tan MX, Le QV. “EfficientNet: Rethinking Model Scaling for Convolutional Neural Networks,“ (in English), *Pr Mach Learn Res*, vol. 97, 2019. [Online]. Available: ://WOS:000684034306026.

[23] Howard AG et al. “Mobilenets: Efficient convolutional neural networks for mobile vision applications,“ *arXiv preprint arXiv:1704.04861*, 2017.

[24] Zoph B, Vasudevan V, Shlens J, Le QV. “Learning Transferable Architectures for Scalable Image Recognition,“ (in English), 2018 *IEEE Conference on Computer Vision and Pattern Recognition*, pp. 8697–8710, 2018, doi: 10.1109/Cvpr.2018.00907.

[25] He KM, Zhang XY, Ren SQ, Sun J. “Identity Mappings in Deep Residual Networks,“ (in English), *Lect Notes Comput Sc*, vol. 9908, pp. 630–645, 2016, doi: 10.1007/978-3-319-46493-0_38.

[26] Simonyan K, Zisserman A. “Very deep convolutional networks for large-scale image recognition,“ *arXiv preprint arXiv:1409.1556*, 2014.

[27] Tan M, Le Q. “Efficientnet: Rethinking model scaling for convolutional neural networks,“ in *International Conference on Machine Learning*, 2019: PMLR, pp. 6105–6114.

[28] Deng J, Dong W, Socher R, Li L-J, Li K, Fei-Fei L. “Imagenet: A large-scale hierarchical image database,“ in *2009 IEEE Conference on Computer Vision and Pattern Recognition*, 2009: Ieee, pp. 248–255.

[29] Zhou B, Khosla A, Lapedriza A, Oliva A, Torralba A. Learning Deep Features for Discriminative Localization,“ (in English). Proc Cvpr Ieee. 2016;2921–9. 10.1109/CVPR.2016.319.

[30] Kingma DP, Ba J. “Adam: A method for stochastic optimization,“ *arXiv preprint arXiv:1412.6980*, 2014.

[31] Pedregosa F (2011). Scikit-learn: machine learning in Python. J Mach Learn Res.

[32] Ho N, Kim YC. “Estimation of Cardiac Short Axis Slice Levels with a Cascaded Deep Convolutional and Recurrent Neural Network Model,“ *Tomography*, vol. 8, no. 6, pp. 2749–2760, Nov 14 2022, doi: 10.3390/tomography8060229. 10.3390/tomography8060229 PMC9680453 36412688

[33] Ho N, Kim YC. Evaluation of transfer learning in deep convolutional neural network models for cardiac short axis slice classification. Sci Rep. Jan 19 2021;11(1):1839. 10.1038/s41598-021-81525-9. 10.1038/s41598-021-81525-9 PMC7815707 33469077

[34] Liew YM et al. “Motion corrected LV quantification based on 3D modelling for improved functional assessment in cardiac MRI,“ *Phys Med Biol*, vol. 60, no. 7, pp. 2715-33, Apr 7 2015, doi: 10.1088/0031-9155/60/7/2715. 10.1088/0031-9155/60/7/2715 25768708

