# Supplementary material for: Evaluation of convolutional neural networks for the detection of inter-breath-hold motion from a stack of cardiac short axis slice images
Source: BMC Med Imaging. 2023 Aug 24;23:113. doi: 10.1186/s12880-023-01070-x (PMC10463654; doi:10.1186/s12880-023-01070-x)
Supplement: Supplementary file 1 — Supplementary Material 1 [file 12880_2023_1070_MOESM1_ESM.docx]

**Supplemental Material**


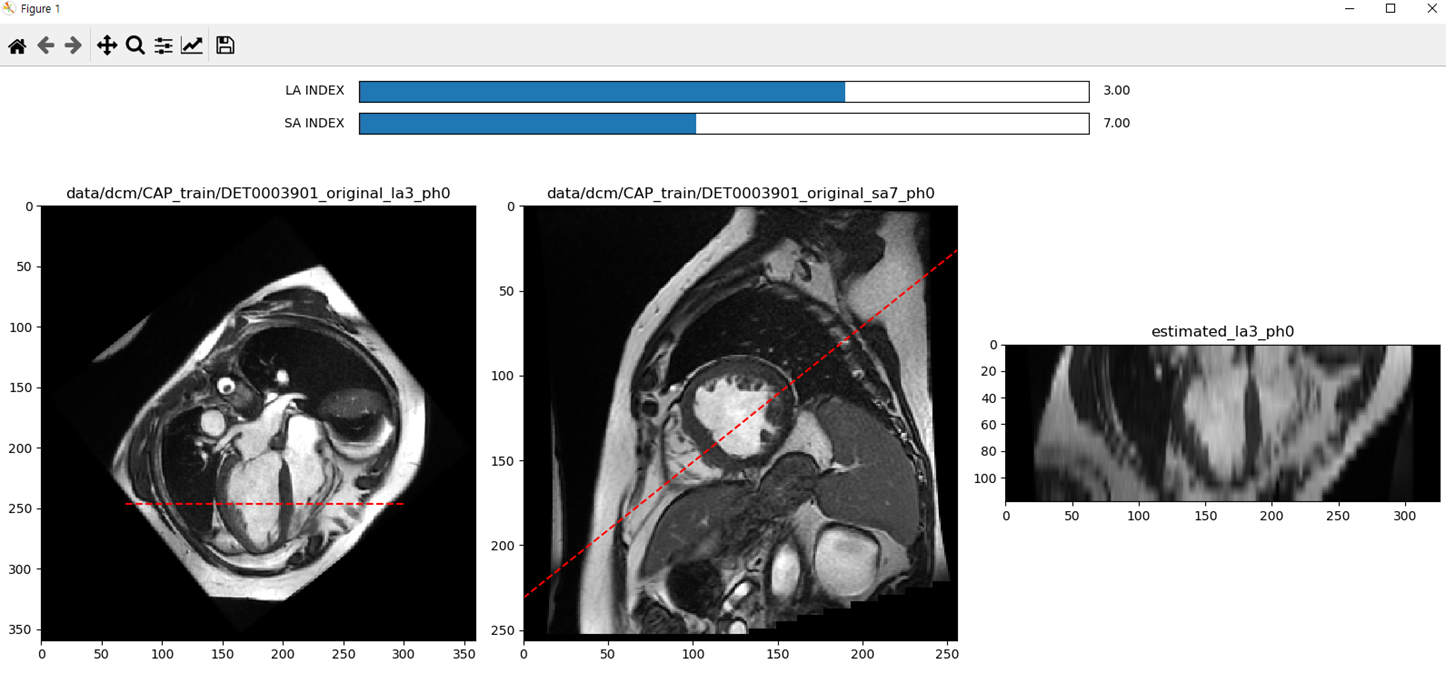


**Figure S1.** A screenshot of the tool. The tool provides visualizations of the intersection line (red dashed line), the long axis slice (left), and the estimated long axis slice (right).


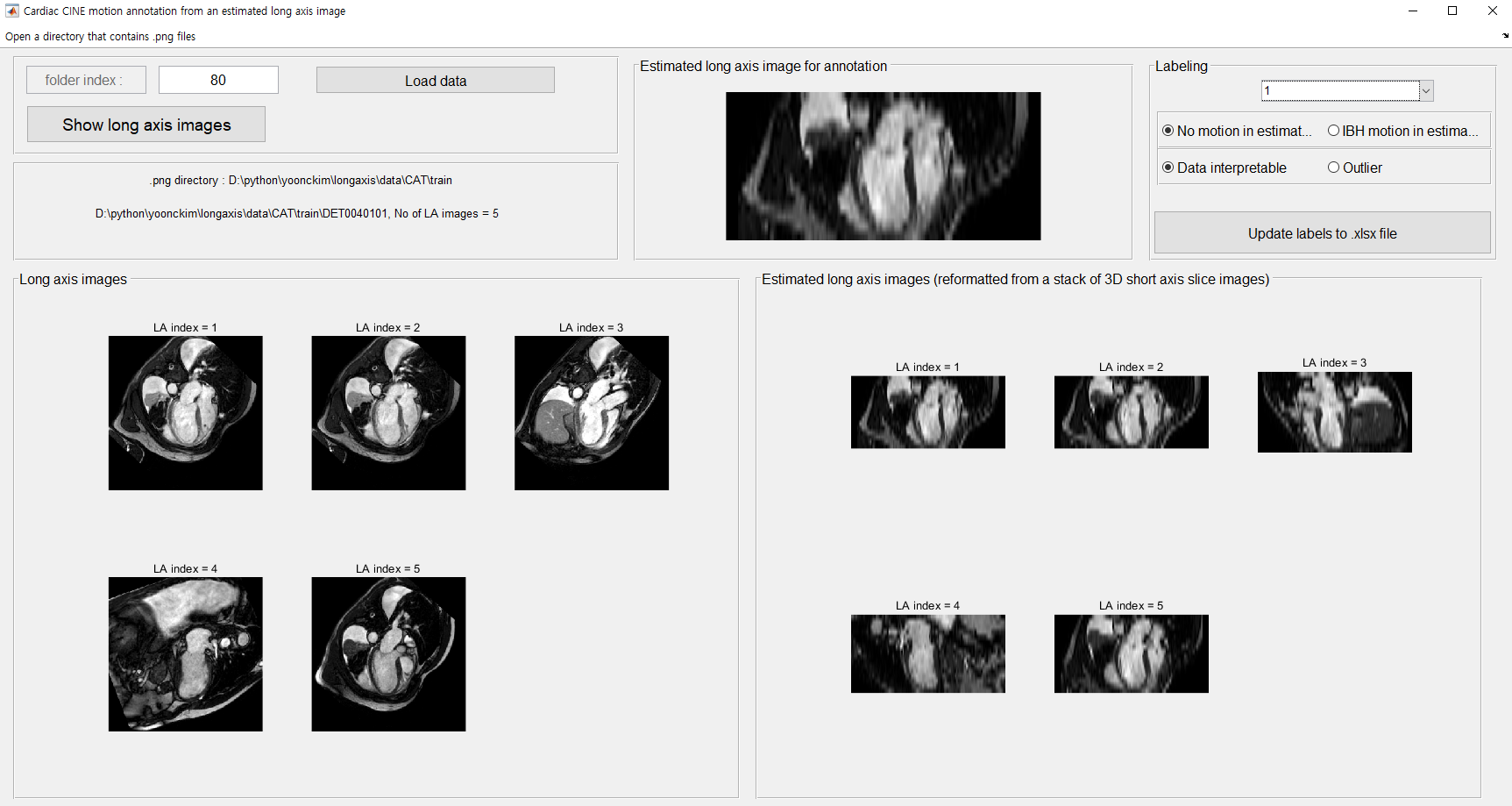


**Figure S2.** A screenshot of the graphical user interface for manual labeling and generation of labeled data for deep CNN classification.

**
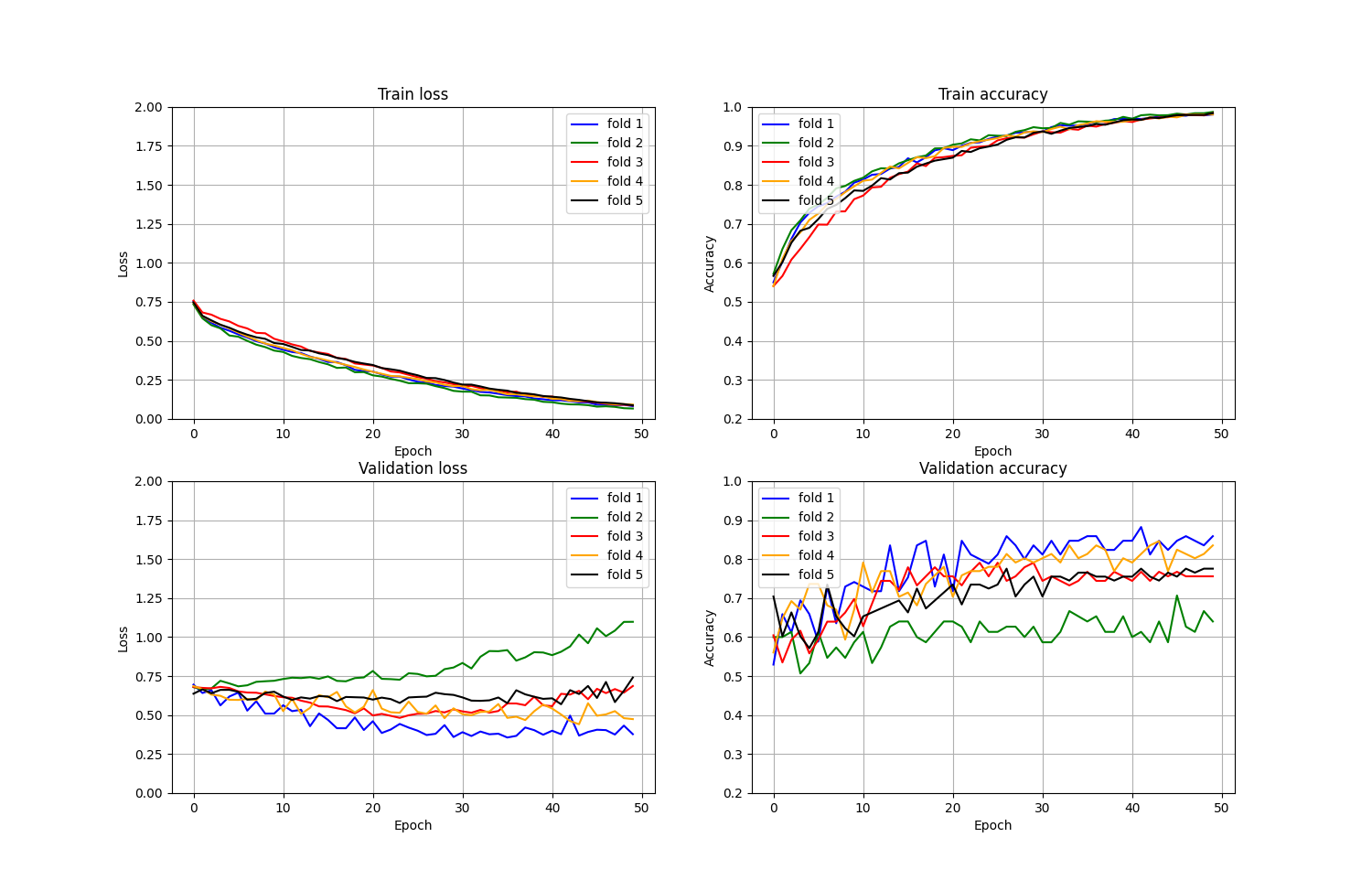
**

**Figure S3.** Training and validation results when using the customized deep CNN model.

**
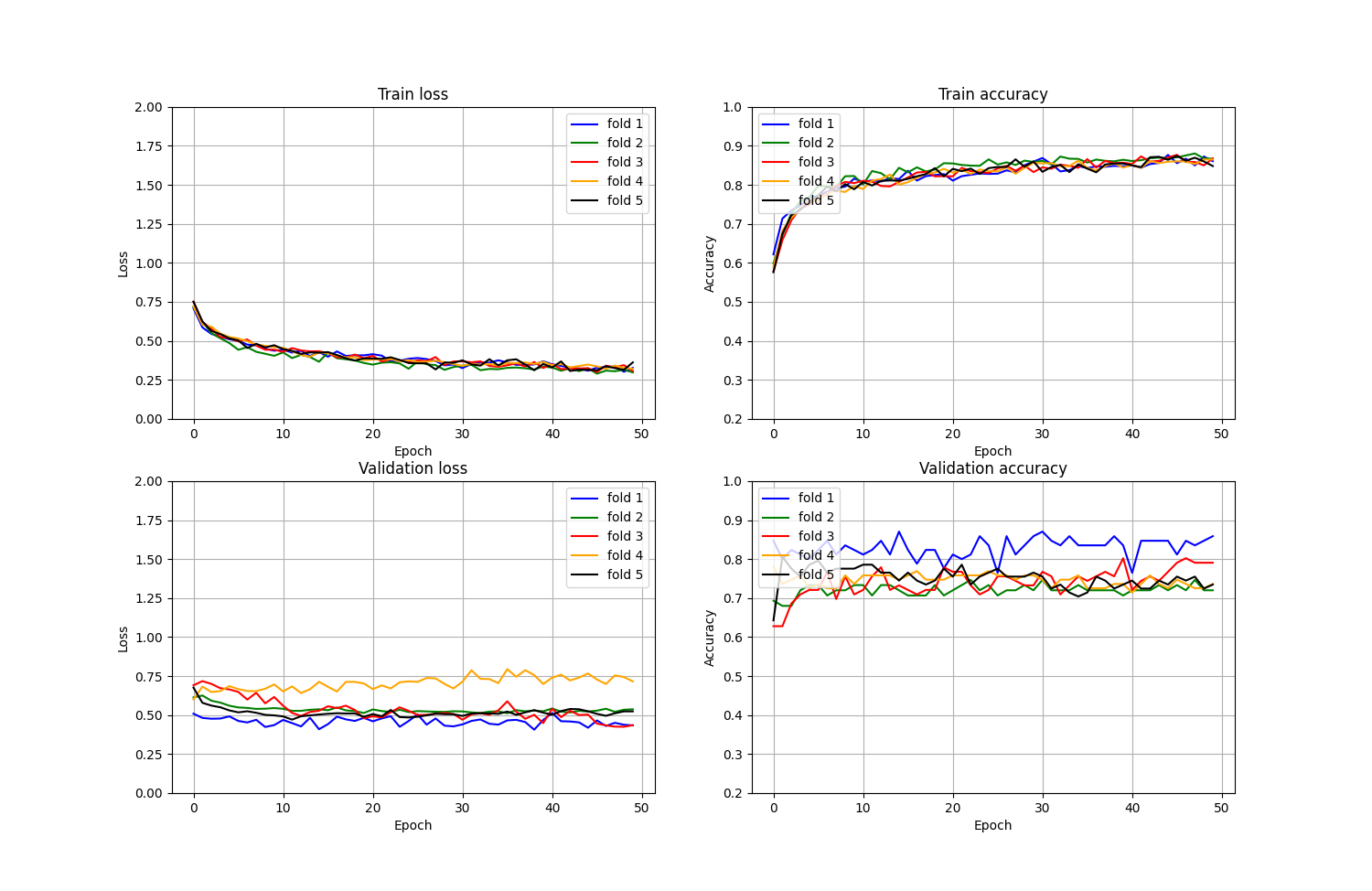
**

**Figure S4.** Training and validation results when using the transfer learning-based model with EfficientNet-B0 as a baseline model.
